# Electromyographical Gait Characteristics in Parkinson’s Disease: Effects of Combined Physical Therapy and Rhythmic Auditory Stimulation

**DOI:** 10.3389/fneur.2018.00211

**Published:** 2018-04-04

**Authors:** Christopher A. Bailey, Federica Corona, Mauro Murgia, Roberta Pili, Massimiliano Pau, Julie N. Côté

**Affiliations:** ^1^Department of Kinesiology and Physical Education, McGill University, Montreal, QC, Canada; ^2^Department of Mechanical, Chemical and Materials Engineering, University of Cagliari, Cagliari, Italy; ^3^Department of Life Sciences, University of Trieste, Trieste, Italy; ^4^Department of Surgical Sciences, University of Cagliari, Cagliari, Italy

**Keywords:** electromyogram variability, electromyogram asymmetry, motor dysfunction, gait, rhythmic auditory stimulation

## Abstract

**Background:**

In persons with Parkinson’s disease (PD), gait dysfunctions are often associated with abnormal neuromuscular function. Physical therapy combined with auditory stimulation has been recently shown to improve motor function and gait kinematic patterns; however, the underlying neuromuscular control patterns leading to this improvement have never been identified.

**Objectives:**

(1) Assess the relationships between motor dysfunction and lower limb muscle activity during gait in persons with PD; (2) Quantify the effects of physical therapy with rhythmic auditory stimulation (PT-RAS) on lower limb muscle activity during gait in persons with PD.

**Methods:**

Participants (15 with PD) completed a 17-week intervention of PT-RAS. Gait was analyzed at baseline, after 5 weeks of supervised treatment (T5), and at a 12-week follow-up (T17). For each session, motor dysfunction was scored using the United Parkinson Disease Rating Scale, and muscle activation amplitude, modulation, variability, and asymmetry were measured for the rectus femoris, tibialis anterior, and gastrocnemius lateralis (GL). Spearman correlation analyses assessed the relationships between dysfunction and muscle activity, and mixed effect models (session × muscle) tested for intervention effects.

**Results:**

PT-RAS was effective in decreasing motor dysfunction by an average of 23 (T5) to 36% (T17). Higher GL activity variability and bilateral asymmetry were correlated to higher dysfunction (ρ = 0.301 −0.610, *p*’s < 0.05) and asymmetry significantly decreased during the intervention (*p* < 0.05).

**Conclusion:**

Results suggest that gait motor dysfunction in PD may be explained by neuromuscular control impairments of GL that go beyond simple muscle amplitude change. Physical therapy with RAS improves bilateral symmetry, but its effect on muscle variability requires future investigation.

## Introduction

In addition to bradykinesia, rigidity, tremor, and postural instability, persons with Parkinson’s disease (PD) commonly experience gait disturbances. These include reduced stride length and walking speed, increased cadence and double support duration (1), freezing (1), and altered activation amplitude of lower limb muscles (2). Dietz and colleagues (3) also observed that persons with PD were less able than healthy controls to modulate their gastrocnemius activation pattern over different walking speeds, suggesting that this may be due to the low activation amplitude. Together, the lower activation amplitude and altered modulation of motor units are believed to be two muscle activation impairments of the electromyogram (EMG) signal associated with Parkinsonian features such as bradykinesia (2, 4, 5).

Motor impairments from PD have been shown to respond to targeted interventions. Levodopa administration, for instance, has been shown to reduce tibialis anterior (TA) amplitude impairments during the heel strike and toe-off phases of gait in persons with PD (6). However, Bloem and colleagues (7) noticed that when persons with PD were on medication, they were actually more likely to suffer a fall. Thus, since gait stability is not improved with levodopa administration (8), other rehabilitation methods are needed to fully address gait-related disturbances (9), and the underlying neuromuscular control mechanisms that would underlie the related benefits need to be identified.

Gait disturbances experienced in PD can be targeted during physical therapy with rhythmic auditory stimulation (PT-RAS) (10, 11). RAS provides consistent auditory cues to assist the regulation of movements during walking, and has been shown to lead to a stride length-mediated increase in gait speed (10, 12). Picelli and colleagues (13) analyzed the effects of RAS on gait patterns in PD and observed that ankle range of motion significantly decreased while maximum hip power during the pull-off phase increased, meaning that there may be a trade-off between the ankle and the hip. Similar results were seen in a kinematic analysis performed by Pau and colleagues (14), who found that a combined PT-RAS intervention increased hip range of motion, with the multi-joint kinematics of persons with PD becoming more similar to those of healthy, age-matched individuals. Together, these results suggest that a new motor strategy develops during PT-RAS, involving increased contribution of proximal (hip) vs distal (ankle) joints. However, further research is needed to understand which muscles and which muscle activity characteristics may be causing the change in motor strategy, and how they are related to the motor dysfunction of individuals with PD.

Studies focusing on lower limb EMG may help identify specific neuromuscular mechanisms implicated in gait impairment and improvement. PD may lower motor unit action potential amplitudes, the summation of which would be recorded by the EMG as lower amplitude bursts of muscle activation (4). During gait, persons with PD have reduced TA and gastrocnemius amplitudes during stance (2, 3, 15, 16) and reduced TA amplitude during swing (2), even after l-dopa administration (2, 16). Cioni and colleagues (2) and Mitoma and colleagues (16) argue that the reductions in activation amplitude may have functional implications for motor control and movement, limiting both the control of foot position and stride length.

In addition to a muscle’s EMG amplitude, its cycle-to-cycle variability and bilateral symmetry may provide additional information on specific characteristics of gait and on the integrity of the underlying neuromuscular control in PD. Kinematic studies have shown that stride-to-stride variability is associated with increased risk of falls (17, 18). This is of particular concern for persons with PD, as this population experiences frequent falls (7, 19, 20) and has a higher risk of falling than similarly aged healthy adults (7). However, individual joint kinematic variability during gait has been shown to not significantly differ between individuals with PD and similarly aged healthy adults (21), suggesting that stride-to-stride variability may instead originate from neuromuscular control deficits that are better studied using EMG. The effects of PD on EMG variability have rarely been studied. In the two known studies, authors assessed the EMG shape variability of the TA and gastrocnemius medialis. While one observed higher EMG shape variability for those with PD than healthy older adults (22), and decreased variability following three weeks of RAS (22), the other did not (10), such that variability characteristics other than signal shape-based may be necessary to elucidate this issue.

Gait asymmetry also seems to have an important functional role in PD. Similar to gait variability, the bilateral asymmetry of gait spatiotemporal and joint kinematic features is consistently higher in persons with PD (21, 23, 24). To our knowledge, only two studies have investigated neuromuscular asymmetry in PD using EMG (10, 22). Miller and colleagues (22) assessed the shape symmetry of several lower limb muscles during gait, finding higher asymmetry of activations for individuals with PD than healthy individuals, and decreased TA asymmetry in a subsample that completed RAS training. However, Thaut and colleagues (10) observed non-significant effects of RAS training on TA and gastrocnemius lateralis (GL) asymmetry, meaning the effects of RAS-based interventions on EMG asymmetry remain unclear.

In summary, some studies have previously quantified the effects of rehabilitation approaches such as PT-RAS on clinical outcomes and on kinematic features of gait in PD. However, none have quantified the effects of the disease and of RAS-based rehabilitation approaches on simple and complex EMG parameters. An exploration of the relationships between patterns of muscle activation and motor dysfunction and the impact of PT-RAS on these parameters is necessary to understand the neuromuscular mechanisms underlying the effects of the disease and of rehabilitation. Therefore, the objectives of this study were to (1) assess the relationships between specific muscle activity patterns and motor dysfunction and (2) quantify lower limb muscle activity pattern changes during PT-RAS. We hypothesized that motor dysfunction would be significantly associated with amplitude, variability, and symmetry characteristics of muscle activity, and that these parameters would be significantly affected by the PT-RAS intervention.

## Materials and Methods

### Participants

As previously described (14), persons with PD were recruited in Cagliari, Italy from the G Brotzu General Hospital, from October 2014 to March 2015. A total of 15 participants voluntarily enrolled into this observational study, and the study had a longitudinal design. Participants were evaluated by an experienced neurologist during the “on-medication” state, 60–90 min after the morning levodopa dose. Inclusion criteria were meeting the UK Brain Bank criteria (25), ability to walk independently, sufficient hearing capacity to detect auditory cues, no significant cognitive impairment (Mini-Mental Status examination > 24; Frontal assessment Battery > 13), absence of dystonia, absence of psychiatric or severe systemic illnesses, absence of any other neurological, cardiovascular and musculoskeletal disorder able to negatively interact with the rehabilitative program, and mild–moderate disability assessed by the modified Hoehn and Yahr (1 ≤ Hoehn and Yahr ≤ 3). Exclusion criteria were any participation in a training or rehabilitative program in the 3 months before the start of the study, and premature dropout before study completion. Ethics approval was received from the local ethics committee (approval number PG/2014/17870), and all participants gave written informed consent in accordance with the Declaration of Helsinki.

### Exercise Intervention

A 17-week PT-RAS program, consisting of a 5-week supervised phase and a 12-week unsupervised phase (14), was administered. During the supervised phase, participants visited the hospital biweekly for 45-min sessions with a certified physical therapist. Targeted exercises for mobility, balance, and posture were completed for 25 min, followed by 20 min of gait training with RAS. Participants were instructed to complete a subset of the exercises and 30 min of gait training with RAS for an additional three times per week in their home. Home exercise duration and activities were recorded in a diary and monitored by the physical therapist. Exercise details can be found elsewhere (14).

Rhythmic auditory stimulation consisted of auditory cues at a pace based on the difference between the participant’s initial gait cadence and that of an age-matched healthy individual (26, 27) as follows:

(1) Below normality: pace was set at 10% higher than the initial gait cadence of a similarly aged healthy adult.
(2) Below and within 10% of normality: pace was set at the normal gait cadence of a similarly aged healthy adult.
(3) Above normality: pace was set at the initial gait cadence of the participant.

During the subsequent 12-week unsupervised phase, participants were instructed to complete the same exercises and RAS daily. Although the training was unsupervised, interviews conducted by a physical medicine physician during the follow-up assessment confirmed that the participants adhered to the program.

### Gait Analysis

Participants visited the gait lab during the “on” state of levodopa three times: pre-program (T0), post-supervision (+5 weeks; T5), and at follow-up (+17 weeks; T17). The process was consistent across visits; a licensed physician completed a motor dysfunction assessment, participants were instrumented for EMG and marker data collection, and then participants completed the gait protocol. The gait protocol was completed without RAS.

Motor dysfunction was assessed by a certified neurologist experienced with PD using the motor examination section of the United Parkinson’s Disease Rating Scale (UPDRS-III). The tool has excellent test–retest reliability (28) and is a clinically relevant measure for persons with PD (29). Supplemental clinical examinations were also conducted by a certified neurologist to further characterize functioning. These included the Tinetti balance assessment tool (Tinetti), the short physical performance battery (SPPB), the Activities-specific balance confidence scale (ABC), and the Freezing of gait questionnaire (FOGQ). The neurologist was blind to the purposes of the study.

Participants were instrumented for both EMG and marker data collection. EMG data were collected using wireless sensors (FreeEMG, BTS Bioengineering, Italy) placed bilaterally on three lower limb muscles: rectus femoris (RF), TA, and GL. The sensors were placed according to SENIAM guidelines (30) and were sampled at 1,000 Hz. Marker data were collected using 22 retroreflective markers placed on the lower limbs and trunk according to Davis’ model (31). Markers were sampled at 120 Hz using an 8-camera optoelectronic motion capture system (Smart-D, BTS Bioengineering, Italy).

Following instrumentation, participants walked barefoot along a 10-m walkway. Participants were instructed to walk at a natural and comfortable speed and participants rested approximately 30 s between trials. A minimum of six walking trials were sampled from each participant.

### Data Reduction

Muscle activation parameters were extracted from the EMG data after identification of gait events. For each leg, the first and second heel strikes during constant-speed walking were identified using a gait analysis software program (Smart-Analyzer, BTS Bioengineering, Italy), and defined gait cycles. Five gait cycles were identified from each leg, and the average gait speed of these cycles was extracted. EMG processing and parameter calculations were completed using Matlab (2011a, The MathWorks Inc., USA). Data were filtered to remove DC bias and underwent bandpass filtering (20–450 Hz second-order, dual-pass Butterworth filter). Linear envelopes were calculated by using a root mean square (RMS) moving-average with a 250-ms window moving forward in 1-ms steps. Linear envelopes were partitioned, resulting in one EMG signal for each gait cycle. Signals were time-normalized to 101 points from 0 to 100%, and amplitude-normalized to their peak value (32, 33).

Data from left and right legs were averaged to compute the following unilateral parameters: RMS, representing the amount of activation per gait cycle, and the modulation index (MI), calculated as the coefficient of variation of the EMG signal from beginning to end of a gait cycle. Muscle activity variability was assessed by taking the cycle-to-cycle coefficient of variation of the RMS (CoV_RMS_) and MI (CoV_MI_) values. Finally, bilateral muscle asymmetry was assessed using the asymmetry index (AI) between the left and right legs for the RMS (AI_RMS_) and MI (AI_MI_) values. AI was calculated using Eq. 1

(1) $$\text{AI}=100-\left(\frac{\text{Leg}1}{\text{Leg}2}*100\right)$$

where Leg1 was the higher value (either the left or right leg) and Leg2 was the lower value (opposite leg); a value of 0 indicated no asymmetry and higher values indicated higher asymmetry.

### Statistical Analysis

All statistical analyses were completed using SPSS (v23, IBM, USA). Data normality was first inspected using Shapiro–Wilks tests. While UPDRS-III did not violate normality, several muscle outcomes did, so Spearman coefficients were computed for the correlation analyses described below. Repeated measures/Friedman’s ANOVAs were conducted to test for effects of session on motor dysfunction and on the supplemental clinical scores.

Correlation analyses were conducted to assess the relationships between muscle outcomes and motor dysfunction. Individual analyses were completed for each session (T0, T5, and T17), as well as for all sessions pooled. Pooled data were visually inspected and when nonlinear relationships were suspected, quadratic curve fitting regressions were computed.

Mixed effect models were conducted to test for effects of PT-RAS on muscle outcomes (34) with unstructured covariance matrices (35). The models tested RMS, MI, CoV_RMS_, CoV_MI_, AI_RMS_, and AI_MI_ for within-participant effects of session (T0, T5, T17) and muscle (RF, TA, GL), as well as interaction effects of session × muscle. Effects of muscle were included to reduce the total number of mixed effect models conducted. Results were confirmed using repeated measures ANOVAs for parametric data, and Friedman’s ANOVAs for non-parametric data. In parametric models, the sphericity was inspected and, when violated, Greenhouse–Geisser corrections were applied (36). For parametric and non-parametric data, *post hoc Bonferroni* tests and Wilcoxon signed-rank tests were conducted, respectively, to identify between-session differences for specific muscles. Since gait speed increased during the PT-RAS intervention (14) and there is evidence that gait speed affects lower limb muscle activity (37, 38), a second group of statistical models were conducted to include gait speed as a covariate. This potential influencing factor did not alter the results; thus, the original models will be reported. Significance for all statistical analyses was set at *p* < 0.05.

## Results

**Figure 1:**
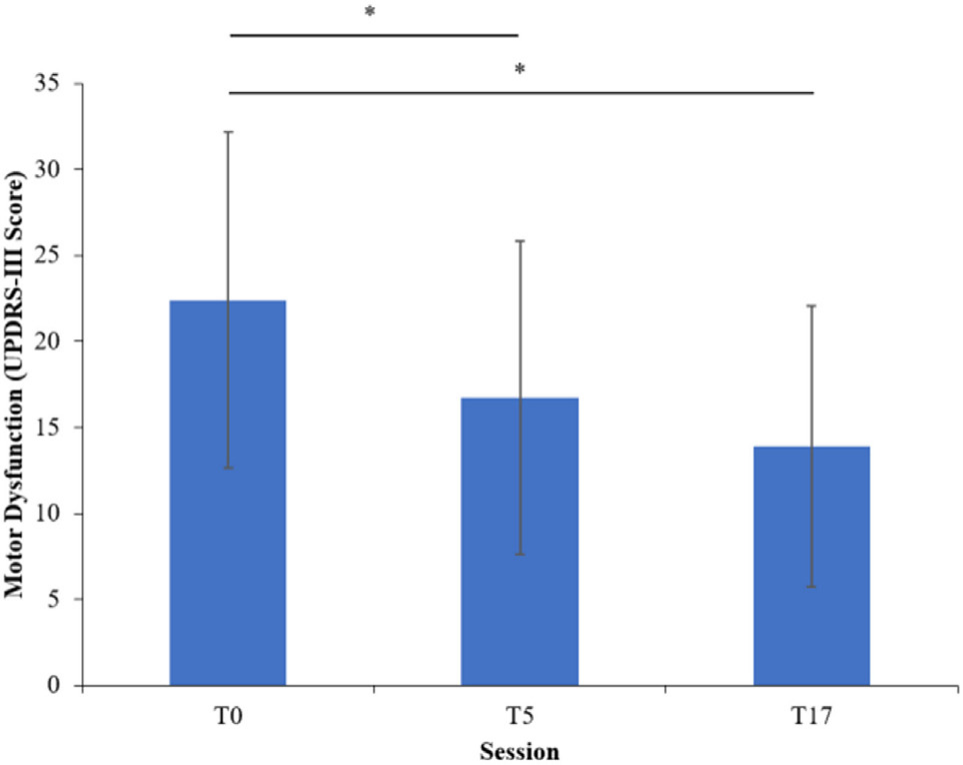
Motor dysfunction at each session of the program. Error bars indicate one SD above and below the mean. Asterisks (*) highlight significant differences (*p* < 0.05).

All aspects of the study were completed by all 15 participants. Their clinical features are shown in Table 1 and their supplemental clinical scores are shown in Table 2. The program was clinically effective, decreasing motor dysfunction UPDRS-III from T0 to T5 by 23% (*p* < 0.05) and from T0 to T17 by 36% (*p* < 0.05) (Figure 1). Using the severity classifications of Robichaud and colleagues (39), group dysfunction decreased from “moderate” (mean score = 22) at T0 to “mild” (mean scores = 17 and 14) at T5 and T17, respectively. Tinetti scores increased from T0 to T5 (*p* < 0.05), ABC scores increased from T0 to T17 (*p* < 0.05), and no session-to-session differences were seen for SPPB or FOGQ (*p*’s > 0.05).

**Table 1:** Features of the 15 persons with Parkinson’s disease (PD) at pre-program (T0), post-supervision (T5), and follow-up (T17).

Participant	PD severity at T0	PD severity at T5	PD severity at T17	Age (years)	PD duration (years)	H-Y	UPDRS-III T0	UPDRS-III T5	UPDRS-III T17
1	Mild	Mild	Mild	68.6	7	1.5	10	8	5
2	Mild	Mild	Mild	56.0	3	1.5	14	6	4
3	Mild	Mild	Mild	79.4	4	2.0	14	6	6
4	Mild	Mild	Mild	74.0	7	1.5	7	5	2
5	Mild	Mild	Mild	75.1	4	2.5	17	15	15
6	Mild	Mild	Mild	79.9	2	1.5	18	10	8
7	Mild	Mild	Mild	74.5	8	1.5	13	7	10
8	Moderate	Moderate	Mild	48.6	2	2.5	29	20	17
9	Moderate	Moderate	Moderate	79.5	15	3.0	24	21	26
10	Moderate	Moderate	Moderate	66.3	6	2.0	28	23	27
11	Severe	Moderate	Mild	71.2	4	2.0	35	22	18
12	Moderate	Moderate	Mild	79.9	5	2.0	29	28	18
13	Moderate	Moderate	Mild	76.9	11	2.0	34	26	19
14	Moderate	Moderate	Mild	69.2	10	2.5	26	22	19
15	Severe	Moderate	Moderate	52.5	0	3.0	38	32	20
Mean (SD)				70.2 (10.2)	6 (4)	2.0 (0.5)^a^	24 (10)^a^	20 (9)^a^	17 (8)^a^

*^a^Median is reported instead of mean*.

*H-Y, Hoehn and Yahr score; UPDRS-III, motor evaluation score on the United Parkinson’s Disease Rating Scale*.

**Table 2:** Supplemental clinical scores of the 15 persons with Parkinson’s disease at pre-program (T0), post-supervision (T5), and follow-up (T17).

Participant	Tinetti balance assessment tool			Short physical performance battery			Activities-specific balance confidence			Freezing of gait questionnaire		
	T0	T5	T17	T0	T5	T17	T0	T5	T17	T0	T5	T17
1	28	28	28	12	12	12	96	96	96	0	0	0
2	21	28	27	6	12	11	48	82	94	14	9	10
3	27	28	28	11	12	12	69	75	84	3	3	7
4	25	26	27	3	9	10				0	0	0
5	27	27	26	12	12	12	84	90	91	9	8	3
6	27	28	28	12	12	12	79	96	99	8	7	5
7	27	27	27	11	12	12	78	85	88	0	0	0
8	26	28	27	11	12	12	74	78	89	0	0	0
9	28	28	28	12	12	12	76	76	91	0	0	0
10	27	28	27	12	12	12	81	83	86	0	0	0
11	27	27	27	12	12	12	89	87	76	5	5	7
12	28	28	28	12	12	12	96	91	98	0	0	0
13	27	26		10	11		92	86		3	14	
14	28	28	27	12	12	12	89	86	73	13	10	12
15	22	26	28	12	12	12	39	72	87	10	5	7
Median (SD)	27 (2)	28 (1)	28 (1)	12 (3)	12 (1)	12 (1)	80 (17)	85 (7)	89 (8)	3 (5)	3 (5)	1.5 (4)

*Empty cells indicate unassessed data*.

**Figure 2:**
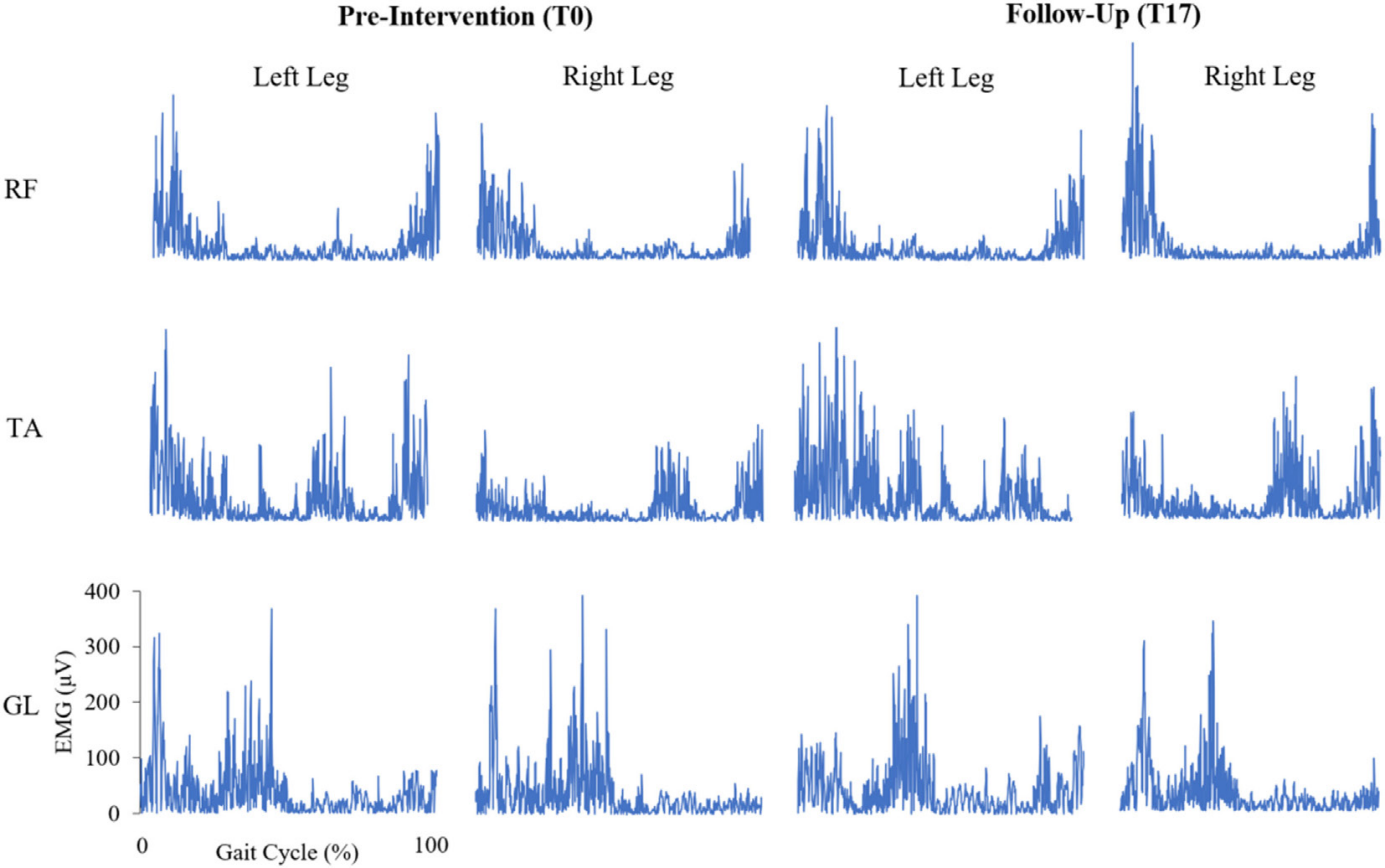
Bilateral, rectified electromyogram (EMG) traces of the rectus femoris (RF), tibialis anterior (TA), and gastrocnemius lateralis (GL) at pre-intervention and follow-up for a representative participant with Parkinson’s disease. *Y*-axes of the EMG traces are scaled consistently across each muscle.

**Figure 3:**
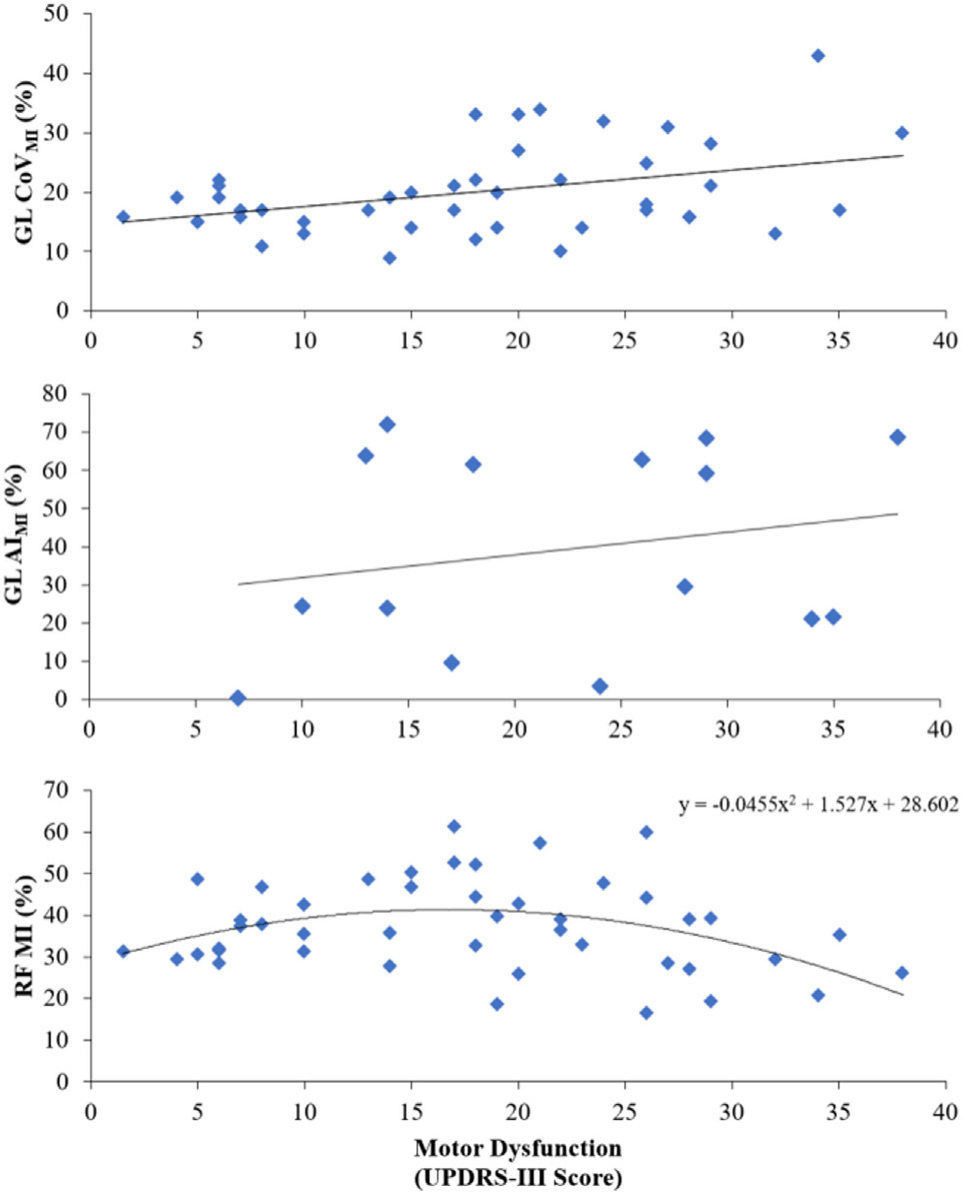
Main relationships seen between motor dysfunction and pooled-session variability of the gastrocnemius lateralis modulation index (GL CoV_MI_), pre-intervention gastrocnemius lateralis modulation index asymmetry (GL AI_MI_), and pooled-session rectus femoris modulation index (RF MI).

Parameters of muscle activation (RMS, MI), variability (CoV_RMS_, CoV_MI_), and symmetry (AI_RMS_, AI_MI_) for each session and muscle are reported in Table 3, and EMG traces of a representative participant are displayed in Figure 2. Results of the Spearman correlation analyses showed that both GL CoV_RMS_ and GL CoV_MI_ were positively correlated to motor dysfunction at T17 (ρ = 0.562, *p* = 0.029; ρ = 0.544, *p* = 0.036). After pooling sessions, only GL CoV_MI_ was correlated to motor dysfunction (ρ = 0.301, *p* = 0.047; Figure 3). This relationship between higher GL variability and higher motor dysfunction was also supported by a correlation between higher GL CoV_MI_ and lower Tinetti score when pooling across sessions (ρ = −0.451, *p* = 0.002). For muscle asymmetry, higher GL AI_MI_ was correlated to higher motor dysfunction at T0 (ρ = 0.610, *p* = 0.016; Figure 3). This relationship between higher GL asymmetry and higher motor dysfunction was also supported by a correlation between higher GL AI_MI_ and lower ABC score when pooling across sessions (ρ = −0.331, *p* = 0.035). Neither RMS nor MI had a linear relationship to motor dysfunction for any muscle.

**Table 3:** Muscle activation root mean square and modulation index (RMS, MI), muscle activation variability (CoV_RMS_, CoV_MI_), and muscle activation asymmetry (AI_RMS_, AI_MI_) of the rectus femoris (RF), tibialis anterior (TA), and gastrocnemius lateralis (GL).

Muscle parameter	Muscle	T0	T5	T17
**Activation**				
RMS (%)^†^	RF	61.2 (9.9)	62.7 (7.4)	60.6 (8.6)
	TA	59.2 (7.6)	61.6 (7.7)	59.0 (6.5)
	GL	53.6 (8.5)	56.4 (6.0)	53.4 (6.2)

MI (%)^†^,^‡^,^a^	RF	36.2 (10.3)	36.2 (10.4)	39.7 (12.2)
	TA	46.6 (10.8)	43.5 (12.3)	48.3 (10.1)
	GL	49.3 (11.1)	44.8 (8.2)	47.2 (9.4)

**Activation variability**				
CoV_RMS_ (%)^†^	RF	11.6 (6.5)	11.9 (5.7)	11.6 (5.9)
	TA	8.3 (3.2)	7.9 (2.4)	9.0 (4.9)
	GL	11.4 (5.8)	9.4 (2.6)	12.0 (4.2)

CoV_MI_ (%)^†^	RF	27.3 (12.4)	26.0 (9.4)	26.0 (7.9)
	TA	17.7 (8.3)	17.4 (6.6)	17.4 (9.3)
	GL	20.8 (9.1)	17.7 (6.3)	21.8 (6.2)

**Activation asymmetry**				
AI_RMS_ (%)^‡^,^b^	RF	38.7 (27.7)	25.9 (18.0)	28.1 (18.2)
	TA	27.2 (15.0)	34.2 (19.7)	35.2 (17.2)
	GL	39.3 (26.3)	26.1 (16.9)	13.3 (10.4)

AI_MI_ (%)	RF	83.9 (129.6)	72.1 (69.9)	44.8 (59.2)
	TA	59.9 (55.7)	41.8 (41.8)	67.0 (49.9)
	GL	138.4 (137.6)	88.3 (85.1)	66.1 (53.0)

*Mean values are displayed for pre-program (T0), post-supervision (T5), and follow-up (T17) sessions*.

*^†^Muscle effect (*p* < 0.05)*.

*^‡^Session × muscle interaction effect (*p* < 0.05)*.

*^a^No *post hoc* differences for the session × muscle interaction effect on MI (*p*’s > 0.05)*.

*^b^T0 < T17 for GL AI_RMS_ (*p* < 0.05)*.

Since a visual inspection of the data revealed a nonlinear trend for RF MI, a quadratic equation was applied. With sessions pooled, there was a significant quadratic relationship between RF MI and motor dysfunction [*F*(2,42) = 4.235, *p* = 0.021, *R*^2^ = 0.168; Figure 3].

The effects of the intervention are reported in Table 3. A significant session × muscle interaction effect was found on MI [*F*(4,14) = 4.1, *p* = 0.021], indicating that the intervention affected the RF, TA, and GL in different ways. However, no significant *post hoc* session-to-session differences were seen for any muscles. A significant effect of muscle was found on RMS [*F*(2,14) = 15.4, *p* < 0.001]. *Post hoc* tests revealed that RMS was significantly lower for GL than both RF and TA, and that MI was significantly lower for RF than both TA and GL. A lack of clear session-based effects indicated that PT-RAS did not alter muscle activation or its modulation.

For muscle activity variability, significant effects of muscle existed for CoV_RMS_ [*F*(2,14) = 7.4, *p* = 0.006] and CoV_MI_ [*F*(2,14) = 11.6, *p* = 0.001]. *Post hoc* tests revealed that the GL was significantly more variable than the TA for CoV_RMS_ and that the RF was significantly more variable than both the TA and GL for CoV_MI_. There were no session-based effects on muscle activity variability, indicating that PT-RAS did not alter muscle activity variability.

However, for muscle asymmetry, a significant session × muscle interaction was found on AI_RMS_ [*F*(4,14) = 4.2, *p* = 0.019]. *Post hoc* tests revealed that GL AI_RMS_ significantly decreased from T0 to T17, revealing a reduction in GL asymmetry from PT-RAS baseline to the final follow-up. No significant session-based effects were found on AI_MI_.

## Discussion

### PT-RAS Intervention Effect on Motor Dysfunction

As shown previously, interventions with RAS resulted in a decrease in PD-related dysfunction (10, 11, 14, 22, 40, 41). The efficacy of PT-RAS is supported by a large amount of empirical evidence [for reviews, see Ref. (42–44)]. It is believed that damage to the basal ganglia compromises movement fluidity and that PT-RAS provides patients with a rhythmic “guide,” facilitating the fluidity of muscular activation. In this regard, an important role would be played by the premotor cortex, an area of the brain important for the elaboration of auditory–motor interactions during complex movements (45, 46). Overall, the mechanisms underpinning the RAS efficacy are still partially unclear. Dalla Bella and colleagues (47) proposed that the malfunctioning of the cortico-subcortico-cortical circuitry may be compensated by the recruitment of “alternative” pathways, such as the cerebello-thalamo-cortical circuitry. Another hypothesis suggested by the authors is that RAS may rely on residual activity in cortico-striatal circuitry.

Although the precise premotor cortex pathways responsible for RAS efficacy remain to be determined, this study showed an average decrease of 36% from pre-intervention to follow-up that resulted in a clinically meaningful shift to milder PD (48). By contrast, Pacchetti and colleagues (49) saw no decrease from the start of their program to their 2-month follow-up. Although their exercises also targeted mobility, balance, and posture, the auditory cues were not restricted to a specific and constant cadence; thus, consistent cueing may be needed to achieve a long-term decrease in dysfunction.

### Relationship of Muscle Patterns to Motor Dysfunction

To our knowledge, this is the first study to show that some lower limb muscle patterns during gait are directly related to motor dysfunction experienced by persons with PD. Higher motor dysfunction was related to higher variability of GL modulation, in line with previous literature describing gait in PD as excessively variable (18, 50). Studies of leg muscle activity variability in young adults suggest that some variability is normal (51, 52), although a specific normality threshold has yet to be identified. Moreover, our sample of persons with PD averaged 70.1 years indicating that age is another factor that could have unique effects on muscle activity variability, as previous studies have shown higher variability in elder groups (53). Finally, higher variability may also be associated with reduced use of gait during everyday activities in those with PD. Whatever the reason, higher variability may be a target of interventions since it has previously been associated with increased risk of falls (17, 18). Moreover, our results specifically target the important role of GL, in contrast to TA or RF, to explain this elevated variability in PD. Given the functional role of GL as an ankle plantar flexor during the push-off phase of gait, interventions to reduce variability could specifically target that phase of gait, as supported by our results.

We also showed that muscle activity asymmetry was related to motor dysfunction before participants began the PT-RAS intervention. Although gait asymmetry is regularly reported in PD (21–24, 54, 55), few studies have reported on correlations between muscle activity asymmetry and disease severity. The linear relationships between GL asymmetry and dysfunction suggest that bilateral neuromuscular coordination deteriorates with more severe PD. Since persons with PD who have severe symptoms, such as freezing, are thought to independently coordinate their limbs (54), and GL is active during the stance-to-swing transition (51) where falls may occur, GL asymmetry may be an important marker of instability-related neurological degeneration from PD.

In our study, those with the lowest and highest dysfunction had the lowest RF MI, meaning that the role of the RF may depend on motor dysfunction severity in a complex way. Robichaud and colleagues (39) also reported severity-specific roles of muscles in the upper limb of individuals with PD. In their study, persons with mild, moderate, and severe PD, as well as healthy controls, completed rapid arm movements, and the duration of the first bicep burst, and its movement-to-movement variability, were calculated. Results showed that persons with mild PD had higher variability in burst duration than both healthy individuals and those with severe PD. Our results could reflect different amounts of rigidity, as persons with mild PD may modulate their RF more as gait automaticity declines, but this may become impaired in severe cases with high rigidity. However, this interpretation is limited by our range of values recorded for the UPDRS-III: the relationship of muscle patterns to motor dysfunction should be further investigated for more severe PD.

### Effect of PT-RAS on Muscle Patterns

Our results showing that the PT-RAS intervention reduced GL muscle asymmetry partially agree with those of others showing decreased muscle activity asymmetry with RAS (22), although the decreases in our study were most prominent at the GL and not the TA as previously suggested (10, 22). In their study, Miller and colleagues (22) enrolled only eight persons with PD in a 3-week RAS-only treatment, and participants ranged in PD severity from moderate to severe (median H-Y = 2.5). Since our sample had milder PD, changes to TA and GL asymmetry could be affected by PD severity. While individuals with PD seem to underuse the ankle joint during gait (14), those with milder PD have been shown to maintain distinct peaks in the ground reaction force profile at the beginning and end of stance (55), in contrast to those with more severe PD (56). Since the TA is activated at the beginning and end of stance (51), it is possible that TA asymmetry is a feature of only more severe cases of PD. Nevertheless, we observed a 66% decrease in GL AI_RMS_ from baseline to follow-up, suggesting an effective role of PT-RAS at least in groups of persons with PD similar to ours.

Despite these promising results of RAS on EMG symmetry, no significant between-session changes were found for muscle activation (RMS, MI) or muscle activity variability (CoV_RMS_, CoV_MI_). Comparing absolute values of muscle activation to previous PD studies was not possible since we followed the recommended procedure of normalizing the activations to their peaks in the gait cycle (32, 33), whereas previous values were not normalized (3). This absence of amplitude normalization may partly explain this absence of between-session change in our amplitude-based parameters, although studies are needed to confirm this.

Similarly, comparing muscle variability across studies is difficult. We selected a simple measure of variability, the coefficient of variation, since it is regularly used to evaluate spatiotemporal gait variability (18, 50). Previous studies have used variability measures based on weighted CoV across increments of the gait cycle (10), and variability of the correlation between individual strides and the ensemble average (22) to show TA and gastrocnemius medialis variability measures of 36.0 and 36.4%, and 15.8 and 18.9%, respectively. Our RMS variabilities were lower compared to both previous studies and our MI variabilities were in line with those of Miller and colleagues (22). However, while they found significant decreases in muscle variability with 3 weeks of RAS, we found no changes to either CoV_RMS_ or CoV_MI_. Together, this strongly suggests that calculation method affects the magnitude and associated findings of muscle activity variability. One advantage to separately assessing CoV_RMS_ and CoV_MI_ was that each may represent a unique feature of PD. This theory is supported by the inconsistent between muscle differences in variability, where CoV_RMS_ was higher for the GL than the TA, but CoV_MI_ was highest for the RF. Only GL CoV_MI_ was related to higher dysfunction, so CoV_RMS_ may not have clinical meaning in PD. Nonetheless, these different calculations of muscle activity variability require a more detailed comparison to evaluate what aspects of PD they measure, and how they respond to changes in motor dysfunction.

Our results have two main implications for the physiotherapy of patients with RAS. First, rehabilitation methods should be enriched with RAS-assisted gait training, given that PT-RAS improves GL symmetry, and this parameter is a significant correlate of motor function. Second, as GL function is associated with less motor dysfunction, physiotherapists should focus on GL-specific rehabilitation. For instance, gradated exercise programs, starting with unweighted calf exercises, could be used to reduce GL activation variability and asymmetry. Knowing that gait in PD is hindered by poorer GL muscle control, this muscle can be isolated in simpler motor tasks, then subsequently developed with gradual exercise programs.

One notable limitation is the low sample size due to the low number of patients available in the hospital and the intensive nature of the 17-week intervention, meaning that the effects of the PT-RAS intervention were not directly compared to those of a control intervention in the same study. We, therefore, caution that both the exercises of the physical therapy program and the RAS training contributed to our muscle activity results, and that their independent effects could not be disassociated. Yet, this study provides the first investigation of gait muscle activity changes during a combined PT-RAS intervention that forms a base for future exploration.

## Conclusion

Our results show that PT-RAS reduced motor dysfunction in persons with PD, and with an unsupervised home program, the improvement was maintained at follow-up. The function of the GL was of importance for the motor function of persons with PD, as higher GL variability and asymmetry were directly related to higher dysfunction. The decreased muscle activity asymmetry may have a causal role in the improved functioning following PT-RAS. The role of muscle activity variability in PD remains unclear, as methodological differences in its calculation may have different meanings for persons with PD.

## Ethics Statement

Ethics approval was received from the local ethics committee (approval number PG/2014/17870), and all participants gave written informed consent in accordance with the Declaration of Helsinki.

## Author Contributions

CB and JC were responsible for study conception, while FC, MM, RP, and MP were responsible for the study organization and execution. CB and JC designed and executed the statistical analyses. CB wrote the first draft of this manuscript, which was reviewed and critiqued by MP and JC. All authors read and approved the final manuscript.

## Conflict of Interest Statement

The authors declare that the research was conducted in the absence of any commercial or financial relationships that could be construed as a potential conflict of interest.

## Abbreviations

PD, Parkinson’s disease; TA, tibialis anterior; GL, gastrocnemius lateralis; RF, rectus femoris; RAS, rhythmic auditory stimulation; PT-RAS, physical therapy with rhythmic auditory stimulation; EMG, electromyogram; UPDRS-III, motor section of the United Parkinson’s disease rating scale; Tinetti, Tinetti balance assessment tool; SPPB, short physical performance battery; ABC, activities-specific balance confidence; FOGQ, freezing of gait questionnaire; MI, modulation index; AI, asymmetry index.
